# Clinical Evaluation of a Novel Tablet Formulation of Traditional Thai Polyherbal Medicine Named Nawametho in Comparison with Its Decoction in the Treatment of Hyperlipidemia

**DOI:** 10.1155/2022/2530266

**Published:** 2022-08-03

**Authors:** Patcharawalai Jaisamut, Channong Tohlang, Subhaphorn Wanna, Acharaporn Thanakun, Thawatchai Srisuwan, Surasak Limsuwan, Wissava Rattanachai, Jarinee Suwannachot, Sasitorn Chusri

**Affiliations:** ^1^Faculty of Traditional Thai Medicine, Prince of Songkla University, Hat Yai, Songkhla 90110, Thailand; ^2^Traditional Thai Medical Research and Innovation Center, Faculty of Traditional Thai Medicine, Prince of Songkla University, Hat Yai, Songkhla 90110, Thailand; ^3^Department of Thai Traditional Medicine, Ban Ta Khun Hospital, Ban Ta Khun, Surat Thani 84230, Thailand; ^4^Department of Thai Traditional Medicine, Singhanakhon Hospital, Singhanakhon, Songkhla 90280, Thailand; ^5^School of Health Science, Mae Fah Luang University, Muang, Chiang Rai 57100, Thailand

## Abstract

In the traditional medical system in Thailand, medicinal plants and polyherbal medicines have been prescribed as lipid-lowering agents, including Nawametho decoction. This polyherbal formulation is described in the Worayokasan scripture. It consists of nine medicinal plants (*Aegle marmelos* (L.), *Carthamus tinctorius* L., *Hibiscus sabdariffa* Linn., *Phyllanthus emblica* L., *Piper longum* L., *Piper nigrum* L., *Terminalia bellirica* (Gaertn.) Roxb., *Terminalia chebula* Retz., and *Zingiber officinale* Roscoe). Apart from its utilization in Thai traditional medicine, there is a lack of evidence supporting its use. This research work thereby aims to formulate and evaluate the tablet containing Nawametho decoction. The feasibility of Nawametho decoction and NawaTab for patients with borderline hyperlipidemia was additionally examined using a prospective, open-label, randomized, parallel-group design. The dry granulation technique was employed to formulate the polyherbal tablets. The tablets were developed using the spray-dried Nawametho decoction as the active ingredient in addition to other excipients. The chosen formulation, the *F*_B_ (NawaTab), consisted of 385 milligrams of the extract, 12% w/w of a diluent (lactose), 8% w/w of a lubricant (magnesium stearate), 5% w/w of a disintegrant (microcrystalline cellulose), and 5% w/w of an anti-adherent (talcum). Their hardness, friability, and disintegration time were 4.4 ± 0.32 kg, 0.05 ± 0.02%, and 4.60 ± 0.05 min, respectively. Accelerated stability study results revealed that NawaTab was stable for six months at 40°C/75% RH and 25°C/60% RH. Even though taking NawaTabs (500 mg twice daily) for eight consecutive weeks was unable to improve the lipid profile of the patients, the administration of Nawametho decoction (30 mL twice daily) was associated with a significant decrease in serum triglycerides of the patients. The results show that the dry granulation technique is suitable for the formulation of NawaTab based on the tablet evaluation. Furthermore, the triglyceride-lowering effect of Nawametho decoction was reported for the first time.

## 1. Introduction

The dramatically increasing prevalence of hyperlipidemia in low- and middle-income countries has become one of the greatest threats to public health [1, 2]. Dyslipidemia with either an elevation of total and low-density lipoprotein (LDL) cholesterol or low high-density lipoprotein (HDL) cholesterol plays a crucial role as a potent risk factor for the progress of cardiovascular diseases (CVDs) [3]. According to the fact sheet distributed by the World Health Organization, CVDs are the leading cause of mortality worldwide, accounting for 31% of the global deaths, and approximately three-quarters of these cases occur in low- and middle-income countries [4]. Even though CVDs can cause a heavy burden on the economies of these countries, this disease can be prevented by controlling behavioral risk factors, including hyperlipidemia which is mainly mediated by unhealthy diet, physical inactivity, and obesity [1–4].

As stated by Adult Treatment Panel III of the National Cholesterol Education Program, nonpharmacologic approaches have been recommended as the initial intervention for patients with hyperlipidemia [5]. Among these, Therapeutic Lifestyle Change (TLC) therapy has been proven by several clinical studies to cause reduction in LDL cholesterol and triglyceride levels by 4 to 30% and elevate the level of HDL by 2 to 14% in hyperlipidemia patients [6–10]. However, patient' responses to TLC varied, and poor adherence to this therapeutic protocol was cited as one of its drawbacks [11, 12]. Furthermore, the administration of statins in patients with elevated serum LDL-C is the first-line pharmacological treatment and effectively reduces total cholesterol (TC) and LDL-C levels [13–15]. However, some adverse events such as muscle-related symptoms, increased liver enzyme transaminase, elevated blood glucose, and glycated hemoglobin levels have been reported [14, 16–18]. As a result, successful hyperlipidemia care with fewer side effects is urgently needed to manage high LDL-C levels and reduce CVD morbidity and mortality.

Although global studies have reported on individual medicinal plants and natural products as a promising tool in controlling hyperlipidemia [19–21], recently, there has been a noticeable increase in hypolipidemic effects obtained from traditional polyherbal medicine/mixtures. Some traditional polyherbal preparations such as Hridayarnava Rasa [22], Danggui-Buxue [23], Shengmai-San [24], and Danshen-Gegen [25] possessed remarkable hypolipidemic effects in *in vivo* and clinical study. Several polyherbal preparations have been prescribed in Thai traditional medicine. A few formulas, such as Benja Amarit [26], Ya-hom [27], Jatu-Phala-Tiga [28], have been scientifically proven to exhibit biological activities related to their traditional use. In the present study, the traditional herbal medicine described in the Worayokasan scripture named Nawametho decoction was chosen because it has been used as an alternative treatment for borderline hyperlipidemic patients at Ban Ta Khun Hospital (Surat Thani, Thailand). The formulation is regularly given as decoction and is made from nine medicinal plants: *Aegle marmelos* (L.), *Carthamus tinctorius* L., *Hibiscus sabdariffa* Linn., *Phyllanthus emblica* L., *Piper longum* L., *Piper nigrum* L., *Terminalia bellirica* (Gaertn.) Roxb., *Terminalia chebula* Retz., and *Zingiber officinale* Roscoe.

Therefore, the current experiment was aimed at formulating and evaluating the physical properties and storage stability of tablets from Nawametho (NawaTab). Moreover, a pilot randomized clinical study was conducted to assess the feasibility of utilizing Nawametho decoction and NawaTab in patients with borderline hyperlipidemia.

## 2. Materials and Methods

### 2.1. Medicinal Plants and Their Pharmacognostic Specification

As described in Table S1, the following nine medicinal plants (2.5 kg each) were procured from a licensed local pharmacy (Farshen Orsot Part., Ltd., Phatthalung, Thailand) and authenticated by the Materia Medica at the Faculty of Traditional Thai Medicine, Prince of Songkla University (Songkhla, Thailand): *Aegle marmelos* (L.) [Fruits/MTM08-01], *Carthamus tinctorius* L. [Flowers/MTM08-23], *Hibiscus sabdariffa* Linn. [Flowers/ARDA18-05], *Phyllanthus emblica* L. [Fruits/MTM08-72], *Piper longum* L. [Flowers/ARDA18-06], *Piper nigrum* L. [Fruits/MTM08-78], *Terminalia bellirica* (Gaertn.) Roxb. [Fruits/MTM08-91], *Terminalia chebula* Retz. [Fruits/MTM08-92], and *Zingiber officinale* Roscoe [Rhizome/MTM08-98]. Each oven-dried cleaned plant part was powdered, passed through 40 mesh, and kept in tight-seal dark containers at 4°C until further use. One batch of each herb was applied for the entire study to avoid batch-to-batch variation. Physico-chemical characteristics of each medicinal plant were conducted according to the protocols described in the Thai Herbal Pharmacopoeia [29, 30].

### 2.2. Preparation and Standardization of Nawametho Decoction

Nawametho decoction was prepared by taking equal proportions of the mentioned medicinal plants and extracted according to the procedure used in Thai traditional medicine. Briefly, Nawametho powder (100 g) was added to a fine muslin container (30 cm^2^) and mixed with 1.5 liters of distilled water. After boiling for two hours, the filtered Nawametho decoction was subjected to spray drying (Buchi Mini Spray Dryer B-290, Switzerland) with aspirator flow of 30 cm^3^/h, extract feeding rate of 3 mL/mi, and inlet and outlet temperatures of 150 ± 1°C and 110 ± 3°C, respectively. An extraction yield of the dried dark, brown-colored powder of Nawametho decoction was 10.33% (w/w; dry weight basis).

A liquid chromatography-quadrupole time-of-flight mass spectrometer (QTOF-MS; Agilent Technologies, Santa Clara, CA, USA) was employed to obtain the qualitative liquid chromatography-mass spectrometric profile of Nawametho decoction as published earlier [28].

### 2.3. Preparation of Nawametho Tablet (NawaTab)

NawaTab, which contains 385 mg of Nawametho decoction per tablet, was prepared by the dry granulation technique described in petty patent number 1903002586. The amount of spray-dried powder was decided based on the extraction yield of the decoction, as previously stated. The patients were given around 37.28 mL of the decoction per day, for a total of 385 mg, according to the prescription. Excipients used in this experiment include lactose, mannitol, corn starch, magnesium stearate, stearic acid, sodium starch glycolate, microcrystalline cellulose, croscarmellose sodium, talcum, and sodium lauryl sulfate and were purchased from PC Drug Center Co., Ltd. (Bangkok, Thailand).

To obtain the extract containing mixture, dried Nawametho decoction was combined in a cube mixer (CMC10, Thailand) with different concentrations of diluent, which is lactose (10, 12, or 15% w/w of dried powder), mannitol (3 or 5% w/w of dried powder), or corn starch (5 or 8% w/w of dried powder), and lubricant which is magnesium stearate (5, 8, or 10% w/w of dried powder) or stearic acid (2, 4, 6, or 8% w/w of dried powder). Each excipient was separately sieved through a 180 *μ*m sieve before being used [31–33].

Slugs (0.5–1 g) were subsequently made from this mixture using a single-punch tableting machine (TDP-6T, China) and compressed at 4–8 kg/cm^2^ with a flat punch of 13 mm in diameter. The oven-dried granule prepared by breaking down the slug was mixed thoroughly with various disintegrants and anti-adherents as described in Table 2S. Subsequently, the obtained powder was recompressed to archive the final tablets (NawaTab). The weight of the tablets was approximately 424 ± 0.01 mg per tablet. The resulting tablets were stored in an airtight container at 25 ± 2°C for further testing.

### 2.4. Physical Properties and Storage Stability of NawaTab

#### 2.4.1. The Flowability of the Powder

The samples taken from the spray-dried Nawametho decoction and the oven-dried granule were subjected to flowability evaluation, indicated by the angle of repose, bulk density, tapped density, compressibility index, and Hausner ratio according to the procedures described in the United States Pharmacopeia and British Pharmacopeia. The fixed funnel method was employed to estimate the angle of repose of the samples. The accurately weighed powder (5 g) was allowed to flow through the funnel positioned at 4 cm from the flat surface. The resulting height (*h*) and radius (*r*) of the powder cone were measured and used to calculate the angle of repose using the following equation: *θ* = tan − 1 h/r. The density parameters were estimated in triplicate using 5.0 g of each powder in a 25 mL measuring cylinder. The cylinder was tapped three times onto a hard surface from the height of 2 cm at 2-second intervals. After manually tapping the cylinder from the height of 3.0 ± 02 cm three times, this volume was recorded as a bulk volume. A tapped volume was recorded when there was no volume change by continuously tapping this graduated cylinder. The values were employed to estimate Hausner's ratio and Carr's compressional index [31–33].

#### 2.4.2. Physicochemical Characterization of NawaTab

According to the United States Pharmacopeia and British Pharmacopeia [31–33], the resulting tablets were determined for their appearance, weight variation, thickness, diameter, and hardness. A tablet hardness tester (Erweka, D-63150, TBH 125, Heusentsamn, Germany) was employed to measure the thickness, diameter, and hardness of NawaTab. The friability of NawaTab was determined using a friabilator (ERWEKA, D-63150, TAR 120, Heusenstamm, Germany) and expressed as the percentage of weight loss of the samples (6.5 g) after rotation at 25 rpm for 4 minutes. The disintegration time was measured on six tablets of NawaTab by the disintegration test apparatus (ERWEKA, D-63150, ZT 222, Heusenstamm, Germany) at 37.0 ± 2°C using distilled water as the disintegration media.

#### 2.4.3. The Stability of NawaTab

Stability tests were performed to assess the physical stability of the formulation under accelerated storage conditions. Samples of NawaTab were stored in an aluminum foil pouch either at a temperature of 25 ± 2°C and relative humidity of 60 ± 5% RH or at a temperature of 40 ± 2°C and relative humidity of 75 ± 5% RH. At the end of six months, samples were taken out and tested for thickness, hardness, friability, and disintegration time. All the tests were done in triplicate, and the findings were presented as mean ± standard deviation [31–33].

### 2.5. An Open-Label, Randomized Pilot Clinical Assessment in Borderline Hyperlipidemic Patients

#### 2.5.1. Design, Sample Size, and Patients

The experiment was carried out as a prospective, open-label, randomized, parallel-group design and conducted for eight weeks at Singhanakhon Hospital (Songkhla, Thailand) from July 2018 to March 2019. The study procedure was performed according to the local laws and the Declaration of Helsinki's statement for medical research involving human subjects and approved by the Ethics Review Committee of the Faculty of Traditional Thai Medicine, Prince of Songkla University (EC62/TTM.02—004). An e-mail was sent to prospective participants informing them of the study's details. The potential participants who expressed willingness and ability to participate in this clinical study were then approached. After signing an informed consent form, each patient was screened and enrolled in the study. During the experiment, all participants had the right to withdraw at any time.

The G^∗^Power program was used to calculate the appropriate sample size for this research, based on a priori statistical power analysis. According to previously published research, the measurement was focused on an estimated difference in blood triglyceride between two groups, an alpha level of 0.05, and a power of 0.78; the sample size was 12 [34, 35].

Participants with the given criteria were included in the present trial: (1) newly diagnosed hyperlipidemia Thai male and female outpatients aged between 20 and 55 years with fasting serum triglyceride of 150–300 mg/dL and LDL-C ≥ 100–160 mg/dL; (2) no medical histories of severe health concerns such as cancers, hypertension, cardiovascular disease, diabetes mellitus, and thyroid diseases; (3) normal test results in the routine physical examination and laboratory tests. Patients taking medication or supplements that might affect lipid absorption and metabolism within the last 12 weeks; having a medical history of allergic reactions to herbs, drugs, or food; and being pregnant or lactating were excluded from this study.

#### 2.5.2. Interventions and Measurement of the Outcomes

For eight consecutive weeks, the eligible patients were given 30 mL of Nawametho decoction or one tablet (500 mg) of NawaTab twice daily, before breakfast and dinner. In addition, the participants were enrolled in a self-management program for hyperlipidemia. The primary outcomes, which are the levels of total cholesterol, low-density lipoprotein (LDL), high-density lipoprotein (HDL), and triglyceride, were determined after fasting for 12 h at the baseline, four weeks, and endpoint (8^th^ week). The levels of FBG and the parameters indicating liver and kidney functions were measured in parallel using the protocols as previously published.

#### 2.5.3. Statistical Analysis

All analyses were performed using the Statistical Package for the Social Sciences program (SPSS 17) for Windows, and the obtained information was expressed as mean ± SD. The Shapiro–Wilk test was used to ensure that the data were distributed normally. The paired *t*-test or Wilcoxon signed-rank test was employed to compare the parameters obtained before and after treatment. The two-tailed independent samples *t*-test or Mann–Whitney *U* test was used to compare groups. Statistical significance was described as a *p*, *P* value of 0.05 or less.

## 3. Results

The first objective of this experiment was to develop the formulation and evaluate the physical properties and stability of traditional polyherbal tablets made from concentrated Nawametho decoction that was prepared by the dry granulation technique. The detailed physicochemical profiles of Nawametho's herbal components are presented in Table 1. Their parameters, including foreign matters, loss on drying, total ash, acid-insoluble ash, and soluble extractive values, comply with either the Thai Herbal Pharmacopeia or the Ayurvedic Pharmacopeia of India.

The flowability parameters (data not shown) of the spray-dried Nawametho decoction and the oven-dried granule were found to be 45.29° and 38.31° for the angle of repose, 0.34 and 0.41 g/cm^3^ for the bulk density, 0.48 and 0.50 g/cm^3^ for the tapped density, 29.21% and 18.92% for the compressibility index, and 1.41 and 1.23 for the Hausner ratio, respectively.

Seven formulas of the Nawametho tablets consisted of 385 milligrams of the polyherbal extract, and 115 milligrams of excipients as described in Table 2S was made. Physical properties, including weight variation, friability, thickness, hardness, and disintegration time of the tablets, were evaluated. Based on these parameters, the *F*_B_ (NawaTab) consisting of 385 milligrams of Nawametho extract, 12% w/w of a diluent (lactose), 8% w/w of a lubricant (magnesium stearate), 5% w/w of a disintegrant (microcrystalline cellulose), and 5% w/w of an anti-adherent (talcum) was chosen as a suitable formulation.

As shown in Table 2, NawaTabs are round in shape and yellowish-brown in color with smooth surfaces and were found to have a low weight variation, fast disintegrating property of 4.60 ± 0.05 min, very low friability of 0.05 ± 0.02%, thickness of 2.6 ± 0.2 mm, and hardness of 4.4 ± 0.32 kg. It should be noted that the physical properties of NawaTabs met the requirements of the United States Pharmacopeia and the British Pharmacopeia. Except for disintegration time, there are no significant differences in friability, thickness, or hardness (Table 2) between freshly prepared NawaTabs and those stored for six months, either at a temperature of 25 ± 2°C and relative humidity of 60 ± 5% RH or at a temperature of 40 ± 2°C and relative humidity of 75 ± 5% RH. In addition, our preliminary results showed that this tablet contains active compounds related to hypolipidemic effects, such as gallic acid, chebulic acid, and ellagic acid (Table 3).

**Figure 1:**
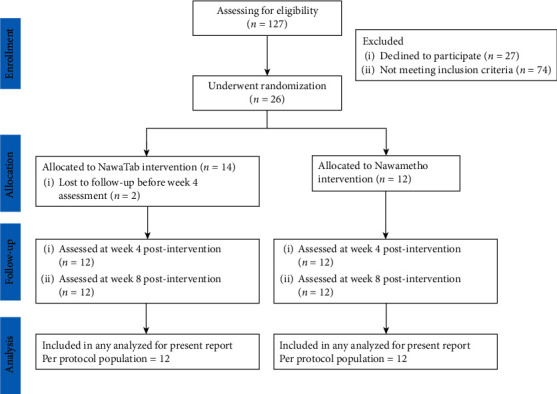
CONSORT flow diagram displaying the progress of the per-protocol population through the 8-week post-intervention visit.

The second objective was to assess the feasibility of utilizing this polyherbal formulation in patients with borderline hyperlipidemia. The CONSORT flow chart in Figure 1 depicts how participants progressed through each point of this pilot. A total of 127 newly diagnosed hyperlipidemic patients were considered for enrollment: 101 did not meet the inclusion criteria, and 26 were randomly allocated to either Nawametho decoction or NawaTab. No statistically significant differences in lipid profiles were found between the two treatment groups at the baseline (Table 4). In a similar manner, at the first and second visits after the 4^th^ and 8^th^ week of the intervention (Table 4), no significant changes in the levels of TC, TG, LDL, and HDL were observed between the two groups.

Furthermore, the administration of NawaTabs (500 mg twice daily) for eight consecutive weeks fails to reduce TC, TG, and LDL adequately. Even though the consumption of Nawametho decoction at 30 mL two times a day caused a significant reduction in the TG level (229.67 ± 68.30 mg/dL (at baseline) vs. 167.50 ± 38.34 (at 8^th^ week)), the levels of LDL were found to be increased significantly. It should be noted that 11 out of 12 (91.67%) of the patients who received Nawametho decoction had improvement in the TG level. In contrast, a decrease in the TG levels was found in 8 participants (66.67%) in the NawaTabs group.

During the 8-week follow-up, there were no adverse events associated with administering Nawametho decoction or NawaTabs among the 24 patients enrolled in this trial. Table 4 shows no significant pattern of changes observed in renal or hepatic functions of the participants in both groups. Based on results obtained, Nawametho decoction may alleviate the level of TG and might be offered as an alternative treatment for the management of hypertriglyceridemia.

## 4. Discussion

The spray-dried Nawametho decoction was prepared as oven-dried granule and employed as an active ingredient in the current experiment to design, develop, and evaluate tablets by the dry granulation. The angle of repose and Hausner ratio of these samples indicating their flow property have been described as follows by the USP 30–NF 25: excellent (25–30° and 1.00–1.11, respectively), good (31–35° and 1.12–1.18), fair (36–40° and 1.19–1.25), and poor (>41° and >1.26) [31, 32]. Therefore, the spray-dried Nawametho decoction and its oven-dried granules exhibited poor and fair flow properties. The improvement in flowability observed in oven-dried Nawametho decoction granules is consistent with previous research [36, 37]. The dry granulation was previously shown to increase the flowability of spray-dried plant extracts, potentially increasing particle size and thereby influencing tablet characteristics of medicinal plants.

Tablets are the most common pharmaceutical oral dosage form because they have many advantages over other dosage forms, including low manufacturing cost, tamper resistance, excellent precision, and minor variability of active ingredients for a unit dose. Appropriate formulations, NawaTab, for direct compression were made. Lactose, magnesium stearate, microcrystalline cellulose, and talcum were used as diluent, lubricant, disintegrant, and anti-adherent agents, respectively. These compounds are frequently employed as excipients in oral dosage form for chemical- and natural-derived active ingredients [38–41]. Magnesium stearate is a low-cost lubricant with a high melting point and chemically stable composition commonly used in the pharmaceutical tableting industry [42]. Similar to our results, several researchers have confirmed that the addition of magnesium stearate improved the flowability of the formulation [42, 43]. It should be noted that the amount of magnesium stearate employed in the present formulation may result in hydrophobic coating on NawaTab, which may cause ineffective drying and wetting of the tablets, resulting in an increase in the time required for the tablet to disintegrate.

Even though the chosen disintegrant, microcrystalline cellulose (Avicel PH 102), was found to produce good mechanical properties, its resulting tablets released the active principle slower than Avicel PH 101 tablets [44, 45]. The disintegration and dissolution behavior of tablets have the most significant impact on a drug's efficiency. NawaTab was categorized as immediate-release tablets intended to completely disintegrate and dissolve within a period of 2.5 to 10 min when exposed to physiological fluids. Microcrystalline cellulose demonstrated its nature at a rapid water-wicking rate with minimal elastic deformation [45]. The tablet disintegration observed in this formulation is possible due to these properties.

The spray-dried Nawametho decoction contains gallic acid and ellagic acid, which have been proven to exhibit hypolipidemic effects in various animal models [46–48]. The preliminary information obtained from the pilot randomized clinical study revealed that the administration of NawaTabs (500 mg twice daily) did not cause reduction in TC, TG, and LDL levels of the patients. Conversely, the consumption of Nawametho decoction showed an improvement in the TG level. It should be noted that NawaTabs is less effective than Nawametho decoction. In contrast to a previous systematic review, the effectiveness and safety between granules and decoctions of Chinese herbal medicine showed no significant statistical differences [49]. Reduction in TC and LDL levels was not observed in this pilot study, but several *in vivo* experiments have confirmed the hypolipidemic effects of Nawametho's herbal components. For example, *H. sabdariffa* [50], *P. emblica* [51], *P. longum* [52], *P. nigrum* [53], and *T. chebula* [54] resulted in the increase of HDL-C level and decrease of TC and TG levels in diet-induced hyperlipidemia rat models. *C. tinctorius* [55, 56], *H. sabdariffa* [57], and *T. bellirica* [58] were additionally reported to inhibit the progression of atherosclerosis and exhibit hypolipidemic effects in other hyperlipidemic animals, including rabbits, hamsters, and mice. Previous studies further confirmed the improvement of the lipid profiles in diabetic animals treated with *A. marmelos* [59], *P. emblica* [60], *T. bellirica* [58], and *Z. officinale* [61]. It is speculated that *in vivo* antihyperlipidemic effects observed from these plants were at least involved in their inhibitory activities on adipogenesis and pancreatic lipase activity [58, 62–64]. Therefore, the hypotriglyceridemic effect recorded in this pilot trial is possibly due to these medicinal plants.

Even though the information obtained from this work will be useful for further conducting prospective, randomized, controlled trials, there were some limitations of this clinical observation that need to be clarified, including the small sample size of 26 patients and the absence of untreated control group. In addition, standardized procedures for the herbal preparation, particularly levels of active ingredients such as lycorine and rosmarinic acid, should be further measured. Moreover, the quantification of active ingredient levels, particularly gallic acid and ellagic acid of Nawametho decoction and NawaTab, needs to be compared to explain the phenomenon observed in this pilot study.

## 5. Conclusion

The present study deals with the formulation and evaluation of physicochemical properties of the tablets made from spray-dried Nawametho decoction. In addition, a pilot clinical trial was carried out to evaluate the feasibility of the use of Nawametho decoction or NawaTab in patients with borderline hyperlipidemia. In summary, seven different formulations consisting of 385 milligrams of the decoction were made with variation in diluent, lubricant, disintegrant, and anti-adherent ratios. The evaluation parameters conducted for the formulation *F*_B_ (NawaTab) comply with the pharmaceutical standards described in the United States Pharmacopeia and the British Pharmacopeia. The consumption of Nawametho decoction (30 mL twice daily) was associated with a significant decrease in serum triglycerides of the participants. However, the administration of NawaTabs (500 mg twice daily) for eight consecutive weeks was unable to improve the lipid profile of the patients. Therefore, factors such as the levels of active constituents of these medicines and their pharmacokinetics studies are being examined to further explain the observed phenomenon.

## Figures and Tables

**Table 1:** Pharmacognostic specifications of herbal ingredients used in the preparation of Nawametho as described in Thai Herbal Pharmacopeia and Ayurvedic Herbal Pharmacopeia.

Medicinal plants	Parameters (%; w/w)				
	Loss on drying azeotropic distillation	Soluble extractives		Total ash	Acid-insoluble ash
		Ethanol	Water		
(1) *Aegle marmelos*	5.93 ± 0.22	11.26 ± 0.67	54.25 ± 0.29	3.03 ± 0.05	0.22 ± 0.01
(2) *Carthamus tinctorius*	5.69 ± 0.07	13.91 ± 0.12	36.20 ± 0.21	6.88 ± 0.04	0.91 ± 0.07
(3) *Hibiscus sabdariffa*	5.08 ± 0.36	11.74 ± 0.16	47.46 ± 0.17	9.79 ± 0.07	0.70 ± 0.04
(4) *Phyllanthus emblica*	4.24 ± 0.03	5.23 ± 0.37	20.70 ± 0.75	3.50 ± 0.04	0.90 ± 0.12
(5) *Piper longum*	8.74 ± 0.08	6.44 ± 0.08	8.14 ± 0.95	4.41 ± 0.11	0.29 ± 0.12
(6) *Piper nigrum*	8.74 ± 0.08	6.44 ± 0.08	8.14 ± 0.95	4.41 ± 0.11	0.29 ± 0.12
(7) *Terminalia bellirica*	8.01 ± 0.19	20.23 ± 0.57	27.89 ± 0.96	3.59 ± 0.19	0.17 ± 0.08
(8) *Terminalia chebula*	7.80 ± 0.23	22.09 ± 0.30	33.13 ± 0.17	3.15 ± 0.02	0.27 ± 0.08
(9) *Zingiber officinale*	7.88 ± 0.03	6.49 ± 0.43	16.86 ± 0.15	9.64 ± 0.35	2.01 ± 0.04

The volatile matters of piper longum and piper nigrum were tested using azeotropic distillation method.

**Table 2:** Physical properties obtained from optimized tablet formulation (NawaTab; *F*_B_) kept for 6 months at normal storage conditions (25 ± 2°C/60 ± 5% RH) and accelerated testing condition (40 ± 2°C/75 ± 5% RH).

Physical properties	Storage time (months)		
0	6	6
(at 25 ± 2°C/60 ± 5% RH)	(at 40 ± 2°C/75 ± 5% RH)
Appearance	Yellowish-brown color with smooth surfaces round in shape	Yellowish-brown color with smooth surfaces round in shape	Dark brownish-yellow color with smooth surfaces round in shape
Thickness (mm)	2.6 ± 0.2	2.6 ± 0.2	2.6 ± 0.1
Hardness (kg)	4.4 ± 0.32	4.8 ± 0.23	5.2 ± 0.15
Friability (%)	0.05 ± 0.02	0.06 ± 0.01	0.07 ± 0.02
Disintegration time (min)	4.60 ± 0.05	25.30 ± 0.03	28.70 ± 0.02

**Table 3:** Active constituents found in NawaTab powder.

Molecular formula	Compounds	Molecular weight	Retention times (min)
C_14_H_12_O_11_	(+)-Chebulic acid	356.04	2.092
C_7_H_6_O_5_	Gallic acid	170.02	2.865
C_10_H_12_O_7_	1-O-galloylglycerol	244.06	3.069
C_27_H_22_O_18_	Pterocaryanin B	634.08	5.170
C_21_H_22_O_11_	2,5,7,4′-Tetrahydroxyflavanone 7-glucoside	450.16	5.584
C_21_H_22_O_10_	(2S)-5,6,7-Trihydroxyflavanone 7-glucoside	434.12	6.687
C_14_H_6_O_8_	Ellagic acid	302.01	7.008
C_27_H_30_O_15_	Scoparin 2″-O-xyloside	594.16	7.146
C_28_H_32_O_16_	Rhamnetin 3-robinobioside	624.17	7.217
C_35_H_32_O_13_	Phylloflavanine	660.19	7.480

**Table 4:** The changes of biochemical parameters in newly diagnosed borderline hyperlipidemia patients who taken either Nawametho decoction or NawaTab.

Parameters^*∗*^	Treatment groups						*P* values^*∗∗*^		
	NawaTab [mean (SD); *n* = 12]			Nawametho [mean (SD); *n* = 12]					
	Day 0	4^th^ wk	8^th^ wk	Day 0	4^th^ wk	8^th^ wk	Day 0	4^th^ wk	8^th^ wk
(i) TC (mg/dL)	243.50 (29.79)	249.00 (32.85)	244.08 (31.90)	233.08 (41.17)	244.00 (44.05)	254.41 (43.88)^#^	0.485	0.756	0.516

(ii) TG (mg/dL)	212.25 (44.49)	181.50 (82.58)	202.67 (110.46)	229.67 (68.30)	199.50 (50.07)	167.50 (38.34)^#^	0.467	0.525	0.686

(iii) LDL (mg/dL)	154.83 (26.03)	163.75 (40.20)	154.25 (23.06)	143.25 (38.96)	161.00 (42.97)^#^	177.25 (44.87)^#^	0.401	0.908	0.129

(iv) HDL (mg/dL)	46.33 (14.14)	48.92 (13.84)	49.33 (13.69)	43.83 (12.63)	43.17 (10.87)	43.41 (9.72)	0.750	0.235	0.326
(v) AST (U/L)	39.58 (16.88)	36.33 (11.80)	42.33 (22.98)	42.50 (10.34)	35.08 (7.91)^#^	39.50 (21.40)	0.311	0.772	0.707
(vi) ALT (U/L)	40.92 (36.15)	30.83 (18.49)	36.33 (29.51)	30.42 (14.14)	31.50 (12.96)	30.00 (11.38)	0.543	0.544	0.795
(vii) TBIL (mg/dL)	0.79 (0.37)	0.70 (0.32)	0.82 (0.32)	0.53 (0.25)	0.62 (0.19)	0.63 (0.20)	0.056	0.439	0.103
(viii) BUN (mg/dL)	11.83 (2.37)	12.33 (2.39)	13.42 (3.15)	12.17 (3.24)	12.75 (2.14)	14.25 (2.63)^#^	0.776	0.657	0.489
(ix) Cr (mg/dL)	0.85 (0.21)	0.84 (0.21)	0.81 (0.22)	0.82 (0.25)	0.82 (0.25)	0.80 (0.30)	0.738	0.826	0.956
(x) GFR (mL/Min/1.73 m^2^)	91.69 (14.48)	93.06 (14.43)	93.13 (13.12)	89.75 (19.07)	89.06 (18.64)	85.82 (20.53)^#^	0.781	0.563	0.310

^*∗*^ALT: alanine aminotransferase; AST: aspartate transferase; BUN: blood urea nitrogen; Cr: creatinine; GFR: estimated glomerular filtration rate; HDL: high-density lipoprotein; LDL: low-density lipoprotein; TBIL: total bilirubin; TC: total cholesterol; TG: triglyceride. ^*∗*^^*∗*^A *p* value ≤0.05 was considered as a statistically significant difference between two groups. ^#^There were significant differences in the observed parameters between baseline and posttreatment.

## Data Availability

The datasets generated during the current study can be obtained from the corresponding author upon request.

## Supplementary Materials

Supplementary Materials Table 1S: safety information and antihyperlipidemic properties of Nawametho's herbal components. Table 2S: composition of Nawametho tablets (NawaTab).
