# Supplementary material for: Clinical Evaluation of a Novel Tablet Formulation of Traditional Thai Polyherbal Medicine Named Nawametho in Comparison with Its Decoction in the Treatment of Hyperlipidemia
Source: Evid Based Complement Alternat Med. 2022 Aug 3;2022:2530266. doi: 10.1155/2022/2530266 (PMC9365582; doi:10.1155/2022/2530266)
Supplement: Supplementary Materials — Table 1S: safety information and antihyperlipidemic properties of Nawametho's herbal components. Table 2S: composition of Nawametho tablets (NawaTab). [file 2530266.f1.docx]

**Traditional Thai polyherbal medicine named Nawametho for hyperlipidemic patients: From tablet formulation development to clinical assessment**

Patcharawalai Jaisamut ^a, b*^, Sasitorn Chusri ^c,**^, Subhaphorn Wanna ^a, b^, Acharaporn Thanakun ^d^, Thawatchai Srisuwan ^a, b^, Surasak Limsuwan ^a, b^, Wissava Rattanachai ^e^, Jarinee Suwannachot ^e^

^a^ Faculty of Traditional Thai Medicine, Prince of Songkla University, Hat Yai, Songkhla 90110, Thailand

^b^ Traditional Thai Medical Research and Innovation Center, Faculty of Traditional Thai Medicine, Prince of Songkla University, Hat Yai, Songkhla 90110, Thailand

^c^ School of Health Science, Mae Fah Luang University, Muang, Chiang Rai 57100, Thailand

^d^ Department of Thai Traditional Medicine, Bantakhun Hospital, Bantakhun, Surat Thani 84230, Thailand

^e^ Department of Thai Traditional Medicine, Singhanakorn Hospital, Singhanakorn, Songkhla 90280, Thailand

*Corresponding author: Faculty of Traditional Thai Medicine, Prince of Songkla University, Hat Yai, Songkhla 90110, Thailand. Tel.: +66 74282722; Fax: +66 74282709; *E-mail address:* patcharawalai.j@psu.ac.th (Jaisamut P.), ** A shared first authorship

**Author Contributions Statement**

The following authors are noted for their respective contribution in this article: **Patcharawalai Jaisamut:** Conceptualization and supervision on the formulation, **Sasitorn Chusri:** Conceptualization and supervision on the clinical trial, **Patcharawalai Jaisamut and Sasitorn Chusri:** finance resources, and writing and revising the manuscript**, Subhaphorn Wanna, Acharaporn Thanakun, Thawatchai Srisuwan, Surasak Limsuwan:** Assisting in the formulation and stability testing, **Wissava Rattanachai and Jarinee Suwannachot:** Conducting the clinical trial and performing the data analysis.

**Declaration of competing interest**

The authors declare that they have no conflicts of interest.

**Acknowledgments**

This research was supported by a grant from the Thailand Research Organizations Network (TRON) through the Agricultural Research Development Agency (Public Organization)-ARDA; Grant number: CRP6105020370.

**Table 1S** Safety information and anti-hyperlipidemic properties of Nawametho’s herbal components

Scientific names LD_50_ ^(references)^ *In vivo* anti-hyperlipidemic effect Animal models tested ^(references)^

[Parts used/Voucher specimen No] (mg/kg) TC TG LDL HDL

1. *Aegle marmelos* (L.); [Fruits/MTM08-01] >5,000 ^(1)^ ND STZ-induced diabetic rats ^(10)^

2. *Carthamus tinctorius* L.; [Flowers/MTM08-23] 5,000 ^(2)^ Hyperlipidemic mice ^(11)^

3. *Hibiscus sabdariffa* Linn. [Flowers/ARDA18-05] 5,000 ^(3)^ Hyperlipidemic hamsters and rats ^(12)^

4. *Phyllanthus emblica* L.; [Fruits/MTM08-72] > 5,000 ^(4)^ ND Hyperlipidemic rats ^(13)^

Fructose fed ovariectomized rats ^(14)^

5. *Piper longum* L.; [Flowers/ARDA18-06] 5,000 ^(5)^ Hyperlipidemic rats ^(15)^

6. *Piper nigrum* L.; [Fruits/MTM08-78] 5,000 ^(6)^ Obese rats ^(16)^

7. *Terminalia bellirica* (Gaertn.) Roxb. 5,000 ^(7)^ ND ND Hyperlipidemia rabbits ^(17)^

[Fruits/MTM08-91] Type 2 diabetic TSOD mice ^(18)^

8. *Terminalia chebula* Retz.; [Fruits/MTM08-92] 5,000 ^(8)^ ND Hyperlipidemia rabbits ^(19)^

Hyperlipidemic rats ^(20)^

9. *Zingiber officinale* Roscoe. 5,000 ^(9)^ ND Dyslipidemia diabetic rats ^(21)^

[Rhizome/MTM08-98]

*LD_50_; Lethal dose 50%: TC; Total cholesterol: TG; Triglycerides: HDL; High-density lipoprotein cholesterol: LDL; low-density lipoprotein cholesterol: STZ; Streptozotocin: TSOD; Tsumura Suzuki Obese Diabetes: ND; No data available

**Table 2S** Composition of Nawametho tablets (NawaTab).

Extract and Excipients (mg) Formulation

F_A_ F_B_ F_C_ F_D_ F_E_ F_F_ F_G_

Nawametho extract 385 385 385 385 385 385 385

Lactose 38.50 46.20 57.75 - - - -

Mannitol - - - 11.55 19.25 - -

Corn starch - - - - - 19.25 30.80

Magnesium stearate 19.25 30.80 38.50 - - - -

Stearic acid - - - 7.70 15.40 23.10 30.80

Sodium starch glycolate (Explotab) - - - 6.40 13.80 - -

Microcrystalline cellulose

(Avicel PH102) 11.70 19.30 29.05 - - - -

Croscarmellose sodium - - - - - 7.80 15.80

Talcum 11.70 19.30 29.05 - - - -

Sodium lauryl sulfate - - - 6.40 13.80 19.50 31.60
